# Handheld Spectral Sensing Devices Should Not Mislead Consumers as Far as Non-Authentic Food Is Concerned: A Case Study with Adulteration of Milk Powder

**DOI:** 10.3390/foods11010075

**Published:** 2021-12-29

**Authors:** Thierry Delatour, Florian Becker, Julius Krause, Roman Romero, Robin Gruna, Thomas Längle, Alexandre Panchaud

**Affiliations:** 1 Société des Produits Nestlé S.A., Nestlé Research, Route du Jorat 57, 1000 Lausanne, Switzerland; romero.roman@gmail.com (R.R.); alexandre.panchaud@rd.nestle.com (A.P.); 2 Fraunhofer IOSB, Fraunhofer Institute of Optronics, System Technologies and Image Exploitation, Fraunhoferstrasse 1, 76131 Karlsruhe, Germany; florian.becker@iosb.fraunhofer.de (F.B.); julius.krause@iosb.fraunhofer.de (J.K.); robin.gruna@iosb.fraunhofer.de (R.G.); thomas.laengle@iosb.fraunhofer.de (T.L.)

**Keywords:** milk powder, adulteration, handheld devices, consumers

## Abstract

With the rising trend of consumers being offered by start-up companies portable devices and applications for checking quality of purchased products, it appears of paramount importance to assess the reliability of miniaturized sensors embedded in such devices. Here, eight sensors were assessed for food fraud applications in skimmed milk powder. The performance was evaluated with dry- and wet-blended powders mimicking adulterated materials by addition of either ammonium sulfate, semicarbazide, or cornstarch in the range 0.5–10% of profit. The quality of the spectra was assessed for an adequate identification of the outliers prior to a deep assessment of performance for both non-targeted (soft independent modelling of class analogy, SIMCA) and targeted analyses (partial least square regression with orthogonal signal correction, OPLS). Here, we show that the sensors have generally difficulties in detecting adulterants at ca. 5% supplementation, and often fail in achieving adequate specificity and detection capability. This is a concern as they may mislead future users, particularly consumers, if they are intended to be developed for handheld devices available publicly in smartphone-based applications.

## 1. Introduction

The motivation behind the practice of cheating consumers with either misleading, low quality, or sometimes hazardous food is driven by the pursuit of financial profit [1]. As people have counted and exchanged goods since prehistoric times, it is reasonable to accept that food fraud is about as old as humanity. With the rise of monetization, as early as 400–200 B.C. in the Roman Empire [2,3] or in the 5th century with cowries spread from India across the African continent [4], the motivation for profit likely became more persistent and fraudulent practices underwent increasing sophistication. The German chemist Fredrick Accum seems to be one of the very first to address the diversity of fraudulent practices in food [5].

Nowadays, highly monetized economies combined with an increasing complexity of the global food supply chain offer a plethora of opportunities for fraudsters to act on either raw materials (adulteration) or finished products (counterfeit). The horsemeat scandal in 2013 exemplifies this context with horsemeat traded and supplied by several operators across Europe for the manufacturing of food products supposed to be made of bovine meat [6,7]. Obviously, the extent of food fraud is difficult to assess, so is the financial damage to food operators and further the possible impact on consumers’ health. Usually, a fraud opportunity is built upon the concept of ‘food fraud triangle’ made of the victims (numerous enough for a significant profit), the fraudster(s), and the lack of guardians. The surveillance by the guardians is essential in maintaining an effective pressure on the fraudsters for preventing the spread of unethical practices. Audits, prediction tools, and quality controls are pivotal activities that need to be rolled out and managed by the guardians, quality control implying the usage of adequate analytical methods. In broad outline, methods of analysis for the control of food authenticity are either restricted to a defined target (possibly several simultaneously) [8,9] or as broad as possible for trying to pick any deviation from the authentic material [10]. The former is blind to any abnormality out of its scope, while the latter might only flag deviations without identification of the cause.

For food operators, adulteration of raw materials is perceived as a sneaky risk because it is related to the complexity of the supply chain together with the volatility of the prices and the smartness of the fraudsters, overall challenging to predict. Therefore, on an operational standpoint, methods that are rapid and applicable upstream in the food chain are preferred; in that regard, vibrational spectroscopy (infrared and Raman) is suitable for testing material authenticity [11,12]. While benchtop instruments based on Fourier transform infrared (FT-IR), near infrared (NIR), or Raman spectroscopies have proven adequate performance in controlling food quality [13,14,15,16], miniaturized devices have gained popularity in the field [17,18,19,20]. Furthermore, the trend for applications with smartphones might give both instrument vendors and food consumers the impression that food fraud control is accessible on a quick check of the product at the grocery store or at home. The objective of the current study is to benchmark performance of a series of spectroscopy-based sensors, used in handheld devices [21], against the detection of adulterants in skimmed milk powder (SMP). SMP was selected because it is an ingredient heavily traded worldwide, thereby exposed to risks of fraud or non-compliance, and due to the possibility to compare performance with benchtop instruments used in milk factories. As models of adulterants, we have selected compounds that mimic proteins [22,23] and a material used as milk powder substitute and carbohydrate replacement [11,24]. We have assessed the capability of the sensors to detect those at low percentage levels, seen as a realistic domain of practice for fraud. Ultimately, we have put the results of the study in the perspective of running technological trends for quality control and expressed recommendations for the release of reliable products on the market.

## 2. Materials and Methods

### 2.1. Samples, Materials, and Reagents

Over 70 samples of skimmed milk powder (SMP) (low, medium, and high heat) were collected in 2017 from 29 suppliers around the world. The collection also includes three skimmed milk powders of buffalo/cow mixes. Samples were kept under vacuum in sealed aluminum-laminated bags and stored at 4 °C. Standards of semicarbazide hydrochloride, ammonium sulfate and cornstarch (unmodified, approx. 73% amylopectin and 27% amylose) were purchased from Sigma-Aldrich (St. Louis, CA, USA).

### 2.2. Standard Additions and Preparation of Dry Blending

Ten diverse SMPs, selected as control group from the collection of 70 SMPs, were separately spiked with three reactive/unstable potential milk adulterants: semicarbazide hydrochloride, ammonium sulfate, and cornstarch. Samples were adulterated at three fraud levels corresponding to 0.5, 5.0, and 10.0% of the total nitrogen content (based on theoretical calculation with chemical formulas), for the *N*-rich adulterants, or to the lactose content (assuming 90% cornstarch being lactose), for cornstarch. In each case, 50 g solutions were prepared by weighing the exact corresponding amount of adulterant and SMP into 100 mL plastic amber jars. Mixtures were homogenized by vigorous shaking followed by 30 min of geometric mixing in a Turbula^®^ shaker (WAB-Group, Muttenz, Switzerland).

### 2.3. Spray Drying and Preparation of Wet Blending

To create the wet-blending adulterated set, a fraction of each spiked sample, along with the 10 control non-adulterated SMPs, were reconstituted and re-spray dried at laboratory scale in February 2018. To that purpose, 30 g of each sample were weighed into 100 mL glass flasks, to which 45 mL of distilled water were added, for a total-solids content of 40%. A few glass beads were used to facilitate dissolution by immediate vigorous shaking. Spray drying was carried out right after on a Büchi mini spray drier B-290 (BÜCHI Labortechnik AG, Flawil, Switzerland) with nitrogen as spray gas. The following recommended operating conditions for SMP were used: inlet temperature = 170 °C, aspirator rate = 100%, rotameter reading = 40 mm (473 L/h), and pump rate = 30%. The outlet temperature and the feed flow obtained under these conditions were 90 °C and 10 mL/min, respectively.

Due to major differences in the physical properties between the original and the re-spray dried powders (the former is light while the latter slightly compacted), the remaining 63 SMPs were also subjected to the reconstitution and the lab-scale spray drying procedure in September 2018. This step completes two full sets of samples, dry and wet blended. Each set comprising 70 cow SMPs, 3 buffalo/cow SMPs, and 90 adulterated cow SMPs (30 per adulterant), for a total of 163 samples per set. Circa 3 g of each sample were placed in small polyethylene Ziplocs (4 cm × 6 cm) before measurement.

### 2.4. Miniature NIR Sensors and Measurements

Eight miniature spectral devices, prototypes, and commercial NIR sensors, were selected for evaluation. The sensors cover different segments of the NIR spectral region using different wavelength selector and detector technologies. Device **A** was based on multichannel optical filtering (Si array detector, wavelength range 740–1070 nm), whilst technology of devices **B** and **C** was grating with a detector InGaAs diode array (wavelength range 900–1700 nm). Devices **D** and **E**, both equipped with a detector InGaAs diode, were based on technologies MEMS DLP (wavelength range 900–1700 nm) and MEMS FT-IR (wavelength range 1300–2500 nm), respectively. Devices **F**, **G,** and **H** were MEMS Fabry–Pérot interferometers equipped with a detector InGaAs diode; wavelength ranges were 1350–1650, 1550–1950, and 1750–2150 nm, respectively. On purpose, manufacturers and models are not disclosed for preventing any brand damage; the objective of the study is to raise areas of improvement that should be considered for future developments in the field. Spectrum acquisition was performed directly on the Ziplocs, i.e., through the transparent polyethylene packaging of the milk samples. Replicate spectra, recorded together in not more than a 20 min time frame, were obtained at 10 different locations of the Ziploc bags, generating a total of 26,080 spectral data files (8 devices × 326 samples × 10 replicates).

### 2.5. Data Processing

Various spectral filters were evaluated to correct for both scattering and additive/multiplicative effects. Combinations of Standard Normal Variate (SNV) and Savitzky–Golay-smoothed derivatives (SG) were tested. Best model performance, as the lowest difference between explained and predicted spectral variance, was in general obtained with only SNV as data preprocessing method.

### 2.6. One-Class Classifier SIMCA Models, Construction and Validation for Non-Targeted Analysis

A multivariate non-targeted model focuses on defining a class of interest, describing as much object variability as possible. One of the most widely used modelling methods for that purpose is Soft Independent Modeling of Class Analogy (SIMCA), applied in this case to a single class (one-class-classifier, OCC). SIMCA is based on Principal Component Analysis (PCA) as multivariate data analysis method. All variables were mean-centered and scaled to the square root of their standard deviation (Pareto scaling). Cross validation was used as validation strategy, using three random groups of ~33% of the sample population each. Optimal size of SIMCA models was determined by the highest predictive cumulative variance and the lowest difference between explained and predictive cumulative variance.

Both, the score distance (SD or Hotelling’s *T*^2^) and the orthogonal distance (OD) were calculated for each sample according to Equations (1) and (2), respectively. The score distance, SD, or in-model distance, is in this case equivalent to the square Mahalanobis distance:

(1) $${\mathrm{SD}}_{i}=T_{i}{}^{2}=\sum_{c=1}^{C}\frac{{\left(\;t_{ic}-\bar{t}\;\right)}^{2}}{s_{tc}{}^{2}}$$

where $t_{ic}$ is the score value of the i th sample in component c, and $s_{tc}{}^{2}$ equals the variance of $t_{c}$. $T_{i}{}^{2}$ is *F*-distributed if corrected to the corresponding degrees of freedom, C and N−C, according to:

(2) $$T_{i}{}^{2}\frac{\left(N-C\right)}{C\left(N-1\right)}\;\sim \;F_{C,N-C}$$

where N is the number of observations in the work set and C is the number of components in the model or the selected number of components. Hence, if $T_{i}{}^{2}$ > $F_{0.05,C,N-C}\;C$(N−1)/(N−C) then observation i is outside the 95% confidence region of the model.

The normalized OD is the residual standard deviation (absolute distance,$\;s_{i}$) divided by the pooled residual standard deviation of the model ($s_{0}$), corrected to the corresponding degrees of freedom:

(3) $${\mathrm{OD}}_{i}=\frac{s_{i}\;}{s_{0}}\;\nu ={\left(\frac{\;\sum_{k=1}^{K}e_{ik}^{2}/\left(K-C\right)}{\sum_{i=1}^{N}\sum_{k=1}^{K}e_{ik}^{2}/\left(N-C-C_{0}\right)\left(K-C\right)}\right)}^{1/2}\;\nu \;$$

where $e_{ik}$ are the residuals of the i th sample for each spectral variable k, *C*_0_ is 1 for centered data (current case) and 0 otherwise. Factor ν is a correction applied to the working set only. Its value is slightly larger than one and it is a function of the number of samples and components.

The model boundary (normalized critical distance, ${\mathrm{OD}}_{critical}$) is calculated from the critical limit of a cumulative *F*-distribution ($F_{0.05,\mathrm{df}1,\mathrm{df}2}$) at a significance level of 0.05, which sets the false adulterated rate at 5%. The degrees of freedom for the numerator (df1, Observations) and denominator (df2, Model) are determined as follows:

(4) $$\mathrm{df}1\;=\frac{\left(100+{\left(K-\mathrm{df}2\right)}^{1/2}-C\right)\;N}{N-C-C_{0}}\;\mathrm{for}\;K\;>\;\mathrm{df}2\;>\;100$$

(5) $$\mathrm{df}2\;={\left[\left(N-C-C_{0}\right)\left(K-C\right)\right]}^{1/2}$$

### 2.7. Orthogonal Partial Least Square Regression Models for Targeted Analysis

Multivariate linear models were created using Partial Least Square regression with Orthogonal signal correction (OPLS). Models were separately built for both blending sets, dry and wet, and for each adulterant based on four concentration levels, including zero (blank SMPs). A total of 40 average spectra (10 SMPs × 4 adulteration levels × 1 average of 10 replicates) were included in each model. In order to factor out the milk natural variability, internal validation across SMPs (Venetian blinds) was used as validation strategy. This means that each SMPs was entirely removed once (all concentration levels, 4 spectra) in a cross validation of 10 iterations (10 SPMs).

General model performance was estimated through population-weighted metrics such as root mean squared errors of calibration (${\mathrm{RMSE}}_{c}$) and cross-validation (${\mathrm{RMSE}}_{\mathrm{cv}}$), according to the following formulae:

(6) $${\mathrm{RMSE}}_{c}=\sqrt{\frac{\sum_{i=1}^{N}{\left(y_{ref,i}-y_{pred,i}\right)}^{2}}{N-2}}$$

(7) $${\mathrm{RMSE}}_{\mathrm{cv}}=\sqrt{\frac{\sum_{i=1}^{M}{\left(y_{ref,i}-y_{pred,i}\right)}^{2}}{N}}$$

where $y_{ref,i}\;$ and $y_{pred,i}$ are the reference and predicted response values of sample i, respectively, *N* is the number of calibration samples, and 2 accounts for the degrees of freedom lost to an OPLS model (1 prediction factor) fit to mean-centered data. In the case of ${\mathrm{RMSE}}_{\mathrm{cv}}$, $y_{pred,i}$ corresponds to the response values predicted on cross validation.

Optimal model size was determined by the number of components at which the RMSEcv stabilized around its averaged minimum, while differing by less than 30% from the RMSEc.

### 2.8. Model Bias Statistical Test

Models were tested for bias using the Elliptical Joint Confidence Region (EJCR) significance test for the true intercept (a) and slope (b) of the univariate linear regression of predicted versus reference values (6):

(8) $$\sum_{i=1}^{N}n{\left(\hat{a}-a\right)}^{2}+2\sum_{i=1}^{N}y_{ref,i}\left(\hat{a}-a\right)\left(\hat{b}-b\right)+\sum_{i=1}^{N}y^{2}{}_{ref,i}{\left(\hat{b}-b\right)}^{2}=2\;\mathrm{MSE}\;F_{\left(2,\nu ;\alpha \right)\;}$$

in this equation $\hat{a}$ and $\hat{b}$ are the estimated intercept and slope of the univariate regression, respectively; a and b are the coordinate axes variables, F is Fisher–Snedecor statistic for 2 and *N*-2 degrees of freedom at 1-*α* confidence level (95%); and MSE is the mean squared error, determined as:

(9) $$\mathrm{MSE}=\frac{1}{\left(N-2\right)}\sum_{i=1}^{N}{\left(y_{pred,i}-{\hat{y}}_{i}\right)}^{2}=\frac{1}{\left(N-2\right)}\sum_{i=1}^{N}{\left(y_{pred,i}-\hat{a}-\hat{b}y_{ref,i}\right)}^{2}$$

where ${\hat{y}}_{i}$ refers to the response values as predicted by the univariate regression.

### 2.9. Limit of Detection

According to International Organization for Standardization [25] the limit of detection (LoD) of an analyte is estimated considering both the probability of falsely declaring it present (α), or error type I, and the probability of falsely declaring it absent (β), or error type II. IUPAC recommends a confidence level of 95% (α = β = 0.05). The LoD can be defined as a function of the standard error of prediction ($\sigma_{PE}$), determined in this case according to the pseudo-univariate approach described elsewhere [26]:

(10) $$\mathrm{LoD}=\Delta_{\left(\alpha ,\;\beta ;\nu \right)}\frac{\;\sigma_{PE}{}_{0}}{\hat{b}}=\Delta_{\left(\alpha ,\;\beta ;\nu \right)}\;\frac{{\mathrm{RMSE}}_{u}\;\sqrt{1+N^{-1}+h_{0}}}{\hat{b}}$$

where ${\mathrm{RMSE}}_{u}$ is the root mean square error of the univariate linear regression, $h_{0}$ is the leverage at zero analyte concentration (for the most conservative estimation of LoD), and $\Delta_{\left(\alpha ,\;\beta ;\nu \right)}$ is the non-centrality parameter of a non-central *t*-distribution with *ν* degrees of freedom (*N-2* for the univariate regression). When *N* is large (>100), like in the current case, $\Delta_{\left(\alpha ,\;\beta ;\nu \right)}$ converges to the sum of two Student’s confidence intervals. The leverage and the ${\mathrm{RMSE}}_{u}$ are determined as follows:

(11) $${\mathrm{RMSE}}_{u}=\sqrt{\frac{\sum_{i=1}^{N}{\left(y_{pred,i}-{\hat{y}}_{i}\right)}^{2}}{N-2}}$$

(12) $$h_{0}=\frac{{\bar{y}}_{ref}{}^{2}}{\sum_{i=1}^{N}{\left(y_{ref,i}-{\bar{y}}_{ref}\right)}^{2}}$$

where $\bar{y}$ is the average reference value in the regression.

Both data pre-processing and model construction were performed with the commercial software SIMCA 14.1 (Sartorius Stedim Biotech S.A., Aubagne, France).

## 3. Results

### 3.1. Outlier Identification and Spectral Quality Assessment

**Figure 1:**
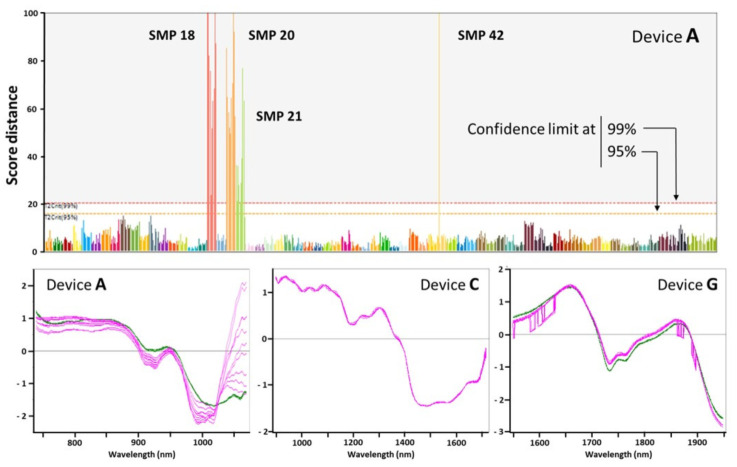
Upper panel: Score distance of the original skimmed milk powder (SMP) samples in a SIMCA model of spectra recorded on device **A** (the 70 SMP samples are color coded with all replicates, *n* = 10, in the same shade). Lower panel: Typical spectral abnormalities observed in devices **A**, **C**, and **G** (normal spectra are green-colored while samples with atypical profiles are in magenta).

All sensors exhibited spectral abnormalities due to either acquisition or internal electromechanical issues. We spotted major spectral abnormalities through unsupervised modelling of the entire data set, with PCA-based methods for instance. In the case of device **A**, with a model based upon eight PCA components explains 99.3% of the spectral variation while predicting 98.6% of it on cross validation, some spectra of four SMP samples fall beyond the limits at 95% and 99% of confidence, thereby are labeled as outliers. Specifically, amongst the spectra acquired with the device **A**, those of SMP 18, 20, most replicates of SMP 21 and one replicate of SMP 42 are obvious outliers (Figure 1). With this particular sensor, the variability among SMPs and the variability among replicates are comparable, highlighting the importance of multiple measuring for a proper assessment of the variance [27,28].

As next step, we compared the outlying spectra to typical ones (within model limits) for gaining insight into the device–sample interaction and the potential causes for each deviation. As regard to device **A**, some spectra exhibited significantly higher absorbance in large spectral regions, most notably around 1500 nm (Figure 1). Given that the outlier replicates show clearly different intensities but with the same overall spectral shape in the same regions, it is suspected that such spectral abnormalities result from uneven powder distribution in the Ziploc containers and/or very large particle size deviations from the collection mean. The spectra from device **C**, on the other hand, showed a high-frequency interference pattern throughout the entire range of wavelengths (Figure 1). Such interferences are usually due to multiple reflections of the light beams on the surface of the sample packaging. This phenomenon was also observed on the devices **B** and **D**. A third type of spectral anomaly was observed on the sensors of devices **F**, **G,** and **H**. A large fraction of spectra exhibited intense step-like absorbance changes. On the device **F**, specifically, these interruptions occurred at fixed wavelengths, making it possible for the SIMCA model to equate the anomalies as irrelevant features. On the other two devices (**G** and **H**), however, the step breaks appeared at random regions and with varying size, as shown in the bottom panel of Figure 1. Such high level of additional noise obscures the natural variability in the sample collection, precluding the extraction of meaningful principal components from the analyzed set.

In general, when anomalies are common to all the spectra acquired with a particular sensor and sufficiently weak to be considered noise, an appropriate cross-validation strategy can render these anomalies non-informative spectral features. In consequence, the outliers will only result from true physico-chemical differences between samples instead of electromechanical device malfunctioning.

### 3.2. Final SIMCA Models and Non-Targeted Analysis

**Figure 2:**
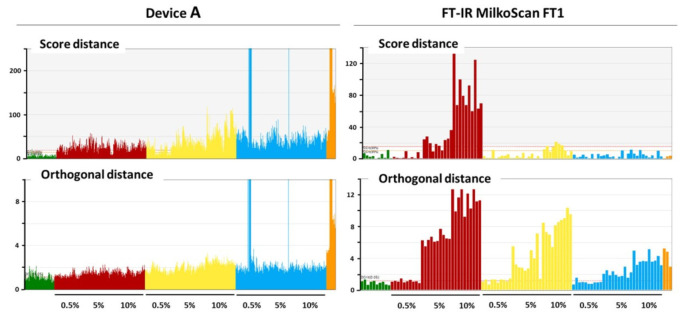
Score and orthogonal distances of control and adulterated dry-blended samples projected onto an OCC SIMCA model of NIR spectra acquired with either device **A** (left panel) or a FT-IR MilkoScan FT1 (right panel) on originally spray-dried skimmed milk powders. Color codes of samples: Green: control; Red: ammonium sulfate-adulterated; Yellow: cornstarch-adulterated; Blue: semicarbazide-adulterated; Orange: buffalo/cow mixtures.

Once the confirmed outliers were removed, we curated the data sets for creating the final OCC SIMCA models, onto which the respective control and adulterated samples were projected in order to assess the overall model sensitivity to milk adulteration. For most sensors, on both dry- and wet-blended sets, a large variability was observed among samples and/or replicates. Most importantly, no response dependence to the adulterant concentration could be distinguished, except for a few ambiguous cases. Specifically, the results for the sensor of device **A** on the dry-blended set of samples do not exhibit a clear change on either distance from left to right for any of the adulterants (Figure 2), except cornstarch, for which a slight increase, most notably in score distance, can be surmised. The rather high variance, however, would make it very unlikely to find significant differences between spiking levels. This will be verified in the next section through linear regression analysis. Some major outliers are also present amongst the spectra of the projected sets of control and adulterated samples. This is a commonality to most sensors due to the spectral aberrations previously described. It is worth highlighting that in the case of the device **A** more than 5% of the control spectra fall beyond the critical orthogonal distance (Figure 2, lower left panel, green), despite the target significance level being established at 0.05. This indicates that the model could not extract the spectral features describing the essence of the studied population. Most likely, this results from the high variance among replicates with respect to the variance among samples, which unavoidably causes part of the noise to be computed into the model, even at optimal model size

**Figure 3:**
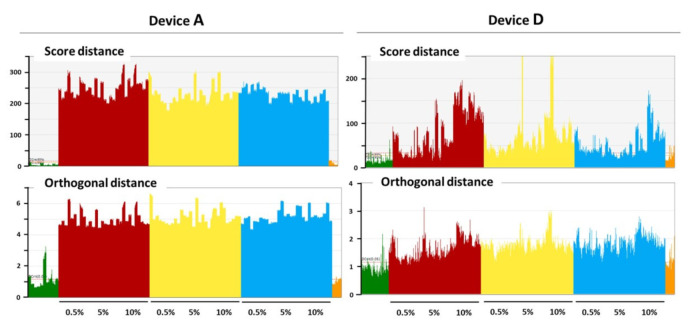
Score and orthogonal distances of control and adulterated wet-blended samples projected onto an OCC SIMCA model of NIR spectra acquired with either device **A** (left panel) or device **D** (right panel) on respray-dried skimmed milk powders. Color codes of samples: Green: control; Red: ammonium sulfate-adulterated; Yellow: cornstarch-adulterated; Blue: semicarbazide-adulterated; Orange: buffalo/cow mixtures.

Regarding the exceptions mentioned earlier, the wet-blended set recorded with device **D** shows an apparent response dependence with the concentration of ammonium sulfate (Figure 3). It is important to underline that this dependence is most noticeable on the score distance (upper panel) instead of the residuals (lower panel), opposite to what is expected for spectral information absent in the modelled population. We obtained a similar pattern with sensor of device **F** (data not shown). This result was observed mostly for the wet-blended set of samples and later confirmed exclusively for ammonium sulfate. Considering that this dependence is most evident on the score distance, it suggests that the response to this adulterant is indirect [29,30], i.e., resulting from changes to major milk properties caused by the ammonium sulfate upon dissolution, and probably further manifested after spray drying.

When compared to laboratory instruments, like the benchtop FT-IR MilkoScan FT1 (mid-infrared, transmission mode, liquid phase, sample homogenized, and temperature controlled), a superior sensitivity to adulteration detection is observed [31]. In this case a clear response-concentration dependence was reported not only for ammonium sulfate, but also for cornstarch and semicarbazide too (Figure 2). Compared to the miniaturized sensors, the OCC SIMCA models from the FT-IR instrument exhibited better general performance. They had reasonable sizes (between four and eight principal components), compatible with the number of major constituents/properties of milk. They also showed very high and close values of explained and predicted variance on cross validation. In addition, the response dependence with adulterant concentration manifested predominantly in the residuals (orthogonal distance), likely due to an adequate sensitivity.

### 3.3. Targeted Analysis and Multivariate Linear Regression Models

Non-targeted methods for food adulteration detection are often complemented with quantitative analysis of the most likely adulterants for the matrix in question. In this study, targeted analysis also serves to confirm the results from OCC SIMCA models, verifying that the technologies under evaluation have the required specificity (unbiased) and sensitivity (detection capability). To that end, we applied multivariate linear regression by partial least squares with orthogonal correction (OPLS) to the data from each sensor on both blending sets.

**Figure 4:**
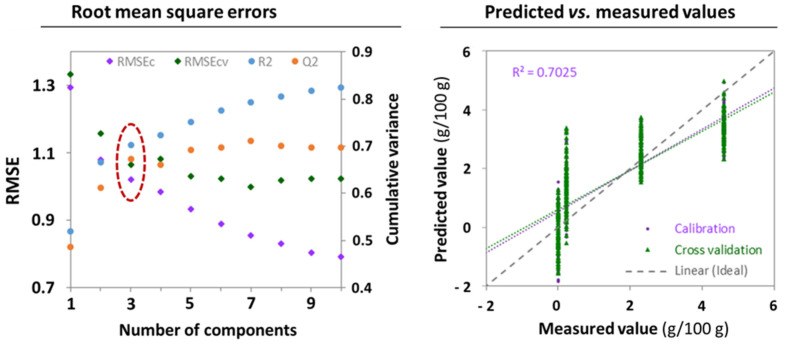
Orthogonal partial least squares regression model for cornstarch in dry-blended skimmed milk powders from spectra acquired with the device **A**: Left panel: Root mean square errors (calibration and cross-validation) and cumulative variances (explained and predicted) as a function of model size (number of total components). Right panel: predicted versus measured values on cross-validation (model fitness).

In the previous section, the OCC SIMCA model for cornstarch, dry blended, from the spectra acquired with the device **A** (Figure 2) suggested not only that SMP adulteration with cornstarch could be detected but, more importantly, that the spectroscopic response increased with the adulterant concentration. The corresponding OPLS model disproved these suggestions (Figure 4). We obtained an optimal OPLS model with three total components, as determined by the first local minimum in the cross-validation error, or the first local maximum in the cumulative predictive variance. Yet, the model exhibited a rather poor linear fitting (Figure 4, right panel; coefficient of determination at R^2^ = 0.703), evidencing a clear bias on both the slope and the intercept respect to the ideal function. These results indicate that the model failed to find spectral features associated to the adulterant; that is, the spectrometer is not sufficiently sensitive to capture the response of cornstarch amongst the signals from the major constituents/properties of the matrix in the tested concentration range. In consequence, interactions between cornstarch and the matrix, or the alteration of its physical properties thereupon, are likely at the origin of the seeming increase in spectroscopic response with the adulterant level.

**Figure 5:**
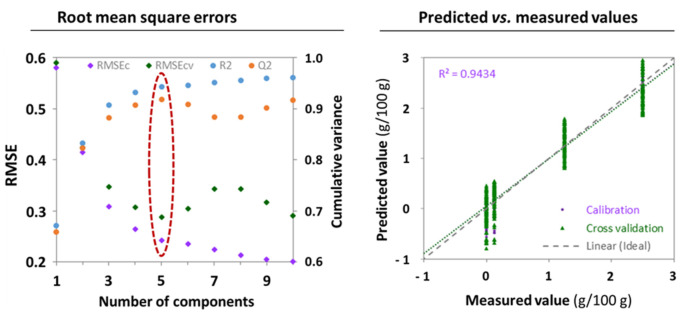
Orthogonal partial least squares regression model for ammonium sulfate in wet-blended skimmed milk powders from spectra acquired with the device **D**: Left panel: Root mean square errors (calibration and cross-validation) and cumulative variances (explained and predicted) as a function of model size (number of total components). Right panel: predicted versus measured values on cross-validation (model fitness).

Conversely, the OPLS model for ammonium sulfate, wet blended, from spectra acquired with the sensor of device **D**, was adequate. First, it showed a more reasonable optimal size of five components (Figure 5), which conforms better to the expected number of major constituents/properties of SMP typically dominating the infrared spectrum, i.e., proteins, lactose, moisture, and particle size. Second, the model managed to find linear combinations of spectral features that relate to the ammonium sulfate concentration, as evidenced by the good R^2^ (0.943, unbiased), and the proximity of calibration and cross-validation predicted values. The LoD was 0.82 g/100 g, corresponding to a fraud level of 3.29% on nitrogen. Although a confident quantification at the target level of 5% *N*-fraud is precluded with this sensitivity (LoQ = 3 × LoD = 9.9% *N*-fraud), the device **D** reliably detects adulteration of SMP with ammonium sulfate when added to the liquid phase (wet blending). Out of the 48 models from the miniature spectrometers (8 sensors × 3 adulterants × 2 blending modes), adequate regressions were obtained for ammonium sulfate only, with devices **D**, **E,** and **F**, confirming the results from OCC SIMCA analysis. Notably, the performance of these devices fails to match that obtained on a benchtop FT-IR instrument^25^, thereby pointing possible technical gaps between emerging miniaturized systems targeting untrained users and benchtop instruments with a long history of proven performance in the laboratory.

## 4. Discussion

**Figure 6:**
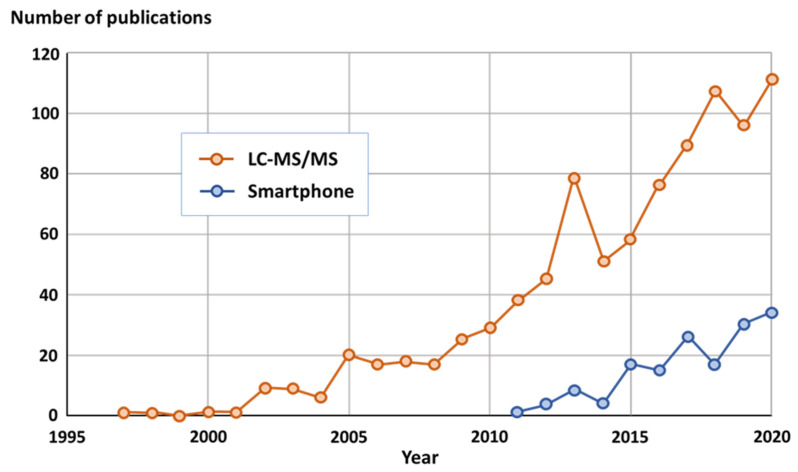
Yearly-based number of publications returned with keywords ‘Food’ and ‘Safety’ in combination with either ‘Smartphone’ or ‘LC-MS/MS’ (LC-MS/MS stands for liquid chromatography-tandem mass spectrometry) on the literature search platform Scopus (www.scopus.com accessed on 15 October 2021).

Exposure to contaminants of chemical origin in food is of concern because they represent either a health risk for consumers or serious economic losses for the business if present as adulterants [32]. Both industry and authority laboratories deploy significant efforts in monitoring these compounds with analytical platforms that provide reliable results, pivotal for a smooth international trade of food. In short, methods of analysis used are either rapid tests for effective support in the operations or hyphenated technologies, usually mass spectrometry-based, that generate comprehensive and confirmatory data. In that regard, liquid chromatography-tandem mass spectrometry (LC-MS/MS) is often considered as a reference and used for validating simple tests [33,34], thereby ensuring the accuracy of those. Smartphone-based sensors that rely on optical detection principles have recently emerged [35,36] (Figure 6), and they represent an attractive technological opportunity that combines rapid testing, connectivity, customization, and portability.

Importantly, the commercialization of tests based on smartphone technologies that enables consumers’ involvement in food testing is an unprecedent step in food quality, while at the same time an opportunity to increase pressure on fraudsters with multiple controls, assuming reliable systems available and easy to operate for people not skilled to the art of chemical analysis. If the latter is not secured, such an opportunity may put the food sector in difficult situations if systems available on the market are not properly validated or do not fit regulatory requirements in terms of limits of detection. Unnecessary concerns from consumers or associations can arise, thereby create damage to food industry, the full sector and ultimately the consumer itself. It worth mentioning that the results of the current study represent the highest expected performance quality wise, as these were obtained under controlled conditions (laboratory). Rather poorer outcomes may be expected in the field or daily life where samples would be analyzed by untrained operators at varying ambient conditions. This raises even more concerns regarding handheld systems, possibly smartphone-based, being publicly available.

We highly recommend research bodies and manufacturers focused on the development of handheld devices for smartphone applications to perform extensive validation for a comprehensive performance assessment. Testing recoveries of the targeted analytes on a couple of samples is insufficient as a validation protocol, and a deep evaluation of the specificity, the ruggedness as well as a benchmark with references mass spectrometry-based methods is of paramount importance for having full confidence in the fitness-for-purpose of the test.

## 5. Conclusions

The results of the study constitute supporting evidence for the lack of sensitivity among the selected miniaturized NIR sensors for reliable detection and quantification of three food adulterants (ammonium sulfate, semicarbazide, and cornstarch) in SMP at 5% of profit or lower. In general, these devices fail to detect the spectral response of the adulterants at the tested levels under a real-world application scenario, i.e., in reflectance, without sample preparation and through the packaging. In the few cases with apparent response–concentration dependence, the relation derived from changes imparted by the adulterant (ammonium sulfate most notably) on major physicochemical properties of skimmed milk upon blending and/or re-spray drying rather than the adulterant itself. In essence, it appears that the sensitivity of these NIR spectrometers is rather insufficient for such rapid screening that might be attractive for operation-driven and consumer-centric applications. Obviously, innovation in the field of miniaturized NIR systems will lead to devices with high levels of performance in the future, and the gap with benchtop instruments might close in some years. In the meantime, the results of the present study encourage caution with regard to possible claims from vendors and manufacturers of miniaturized spectroscopic systems that may communicate outstanding capabilities in checking food quality and authenticity on the go (e.g., while grocery shopping or in a warehouse) and in a fast manner (e.g., with a single measure). Usage for other suggested applications requiring much lower detection capabilities, such as chemical contaminant testing in the µg/kg (ppb)–mg/kg (ppm) range, should be considered even more cautiously.

## Data Availability

The datasets generated for this study are available on request to the corresponding author.
